# Study of the Influence of Desert Sand-Mineral Admixture on the Abrasion Resistance of Concrete

**DOI:** 10.3390/ma18020446

**Published:** 2025-01-19

**Authors:** Aoli Cao, Yuwei Ma, Zhiqiang Li, Xixian Du, Gang Li, Aiqin Wang

**Affiliations:** 1 College of Water Conservancy & Architectural Engineering, Shihezi University, Shihezi 832000, China; aolicao@stu.shzu.edu.cn (A.C.); zhiqiangli2023@163.com (Z.L.); xxdu@stu.shzu.edu.cn (X.D.); wangaiqin@shzu.edu.cn (A.W.); 2 Key Laboratory of Cold and Arid Regions Eco-Hydraulic Engineering, Xinjiang Production & Construction Corps, Shihezi 832000, China

**Keywords:** desert sand, concrete, orthogonal experiment, mechanical properties, abrasion resistance

## Abstract

The incorporation of desert sand-mineral admixture improves the abrasion resistance of concrete. To prolong the service life of assembled concrete channels and mitigate the depletion of river sand resources, the effects of fly ash (FA), silica fume (SF), desert sand (DS), and basalt fiber (BF) on the mechanical properties and the abrasion resistance of concrete were examined, alongside an analysis of their microstructures to elucidate the underlying mechanisms of influence. The results indicated that the abrasion resistance strength of concrete mixed with 10% FA and 0.05% BF alone increased by 80.19% and 81.59%, respectively, compared with ordinary concrete (OC). When SF was added to the concrete at a dosage of 10%, it improved the mechanical properties and the abrasion resistance of the concrete. Furthermore, adding SF resulted in a 12.50% increase in compressive strength and a 12.27% increase in abrasion resistance strength compared to OC. The addition of DS did not significantly enhance the concrete’s abrasion resistance. The combination of ingredients for desert sand concrete (DSC) that provides excellent abrasion resistance was determined using an orthogonal experiment. The optimal mixture consisted of 10% FA content, 10% SF content, 40% DS content, and 0.05% BF content, which increased the abrasion resistance strength by 112.95% compared to OC. Through microscopic analysis, it is found that the width of the interfacial transition zone (ITZ) is an important factor in determining the abrasion resistance of concrete, and a narrower ITZ enhances the concrete’s abrasion resistance. The study’s findings could function as a theoretical reference for the engineering design of DSC.

## 1. Introduction

An assembled channel refers to a precast concrete canal system structure that is initially manufactured in a factory, then transported to the construction site and assembled using mechanized and information-based engineering techniques. It possesses the attributes of high productivity, energy efficiency, environmental friendliness, and more [1,2]. Nonetheless, the rapid development of assembled channels in the field of agricultural water conservancy projects has escalated the demand for sand, resulting in a scarcity of river sand resources, which has driven up prices, while the over-extraction of river sand has adversely affected the ecological environment [3]. Furthermore, the desertification land in the Xinjiang region comprises nearly 30%, and annual precipitation measures approximately 150 mm. This results in frequent dusty weather and significant grassland degradation. Consequently, the rivers and water conveyance channels in Xinjiang exhibit elevated sand content, with sediment concentrations in part of the northern Xinjiang river peaking at 454 kg/m^3^ [4]. The channels are highly susceptible to scouring abrasion and cavitation damage from entrained suspended particle flows [5]. Abrasion damage has emerged as a primary factor contributing to the performance degradation of assembled concrete channels. Consequently, addressing the deficiency of river sand and enhancing the abrasion resistance of concrete is essential for ensuring safe operation and extending the service life of assembled concrete channels.

Deserts encompass 80 percent of the north-western region of China, with Xinjiang, Inner Mongolia, Gansu, and Ningxia being the primary areas rich in desert resources. Hence, the utilization of desert sand (DS) as a substitute for natural river sand can effectively reduce the environmental burden and address the lack of river sand resources. This holds immense importance for safeguarding the ecological environment and advancing the progress of novel construction materials. Currently, numerous scientists have made significant progress in the research of desert sand concrete (DSC). Liao et al. [6] conducted a study where they substituted DS for artificial sand as the fine aggregate in concrete. They examined the impact of the desert sand replacement rate (DSRR) on the mechanical properties, durability, and volumetric stability of the concrete. The findings revealed that as the DSRR increased, there was a general decrease in the concrete’s compressive strength, chloride ion penetration resistance, and frost resistance. Qiao et al. [7] constructed a machine-learning model to forecast the impact of DS and fibers on the freeze–thaw (F-T) damage capacity of concrete. They discovered that substituting river sand with DS enhances the frost resistance of concrete, and incorporating basalt fiber (BF) additionally diminishes F-T damage. Li et al. [8] examined the shear behavior of DSC beams and suggested a revised formula to enhance the accuracy of shear strength prediction. Shen et al. [9] examined the impact of DSRR on the compressive strength of concrete under elevated temperatures. The findings indicated that DSC exhibits excellent high-temperature resistance and superior compressive strength at 20% and 40% DSRR in comparison to ordinary concrete (OC). Liu et al. [10] discovered that the splitting tensile strength, compressive strength, and flexural strength of DSC subjected to high-temperature treatment attained their highest values at a DSRR of 40%. Kaufmann [11] proposed a binder composed of calcium sulfoaluminate cement (CSA) and gypsum mixed with artificial DS, which has been demonstrated to enhance the mechanical properties of concrete and decrease drying shrinkage. Recent studies have demonstrated that replacing concrete fine aggregates with DS not only satisfies engineering criteria in terms of workability, mechanical properties, and durability of DSC but also yields significant financial advantages.

There are multiple methodologies available to improve the abrasion resistance of concrete. These include enhancing the strength of the cement paste [12,13], incorporating supplementary cementitious materials [14,15], utilizing high-hardness coarse aggregates [16,17], optimizing the surface protection technology of concrete [18,19], and enhancing the curing conditions [20]. Research shows that silica fume (SF) is extensively utilized to boost the abrasion resistance of concrete [21,22,23]. However, SF possesses a high level of volcanic ash activity [24], requiring careful consideration of the SF dosage. Excessive SF doping induces concrete volume contraction, leading to cracks forming [25]. Some scholars have investigated the drawbacks of SF and discovered that adding fly ash (FA) [24,26,27] and fibers [28,29] to SF concretes may efficiently reduce concrete shrinkage and decrease crack development, hence improving the concrete’s abrasion resistance.

In conclusion, both DS and mineral admixtures (FA, SF, and BF) can independently improve the mechanical properties of concrete; however, there is insufficient research on the simultaneous formulation of DSC with DS and these mineral admixtures, and the impact of combining these four materials on the mechanical properties, as well as the abrasion resistance of concrete, remains ambiguous. Therefore, in this study, Gurbantunggut Desert sand in Xinjiang, China, was chosen to create concrete. The study examined how the addition of DS, FA, SF, and BF individually affected the mechanical properties and abrasion resistance of the concrete. The specimens were analyzed using scanning electron microscopy (SEM) and X-ray diffraction (XRD) to examine them at a microscopic level. Ultimately, the most optimal DSC mix ratio, which exhibited excellent abrasion resistance, was determined through the use of an orthogonal experiment. This study can enhance the efficient use of DS resources, serve as a reference for the application of DSC in the assembled channels domain, and offer data support for the implementation of DS in engineering and construction in deserts and adjacent regions globally.

## 2. Materials and Methods

### 2.1. Materials

(1) Cement: The cement utilized was P·O 42.5 ordinary Portland cement manufactured by China Xinjiang Tianneng Cement Co., Ltd. (Shihezi, China). The chemical composition and specific surface area of the cement may be found in Table 1.
(2) Fine aggregate: medium sand, fineness modulus 2.7.
(3) Coarse aggregate: The coarse aggregate consists of stones sourced from Shihezi City, Xinjiang Province, China, and has a consistent size grading of 5–20 mm.
(4) Gurbantunggut Desert sand: Located in the Xinjiang region of China (Xinjiang Province, China), the Gurbantunggut Desert is characterized by an average grain size of 0.183 mm and a fineness modulus of 0.334. The chemical composition of the sand is provided in Table 2.
(5) Fly ash: The study utilized the processed class Ⅰ fly ash from China Xinjiang Yue Longda Building Materials Co., Ltd. (Shihezi, China), with its specifications outlined in Table 3.
(6) Silica fume: The study utilized the silica fume manufactured by China Henan Platinum Run Casting Materials Co., Ltd. (Gongyi, China), with the particular characteristics indicated in Table 4.
(7) Basalt fiber: The study utilized short-cut basalt fiber from China Zhengzhou Dengdian Basalt Co., Ltd. (Zhengzhou, China), which had a length of 18 mm. The performance characteristics of the fiber may be found in Table 5.
(8) Water-reducing agent: The study utilized a polycarboxylic acid water-reducing agent manufactured by China Xinjiang Changhong Admixture Co., Ltd. (Shihezi, China), which has a water-reducing efficiency of 20%.
(9) Water: The water utilized in the test was tap water sourced exclusively from the China Shihezi region (Shihezi City, Xinjiang Province, China). The water hardness measured 276 mg/L, while the pH level was 7.4.

### 2.2. Mix Proportion

The concrete had a design strength class of C50 and a water-binder ratio of 0.39. Considering the outcomes of the prior test and the costs associated with the assembled channel, the FA admixture is chosen in proportions of 10%, 20%, and 30%; the SF admixture is chosen in proportions of 5%, 10%, and 15%; the DSRR is chosen in proportions of 20%, 40%, and 60%; and the BF accounts for 0.05%, 0.10%, and 0.15% of the concrete volume. FA, SF, and DS are denoted as a mass fraction, with FA and SF substituting cement in equivalent proportions and DS replacing fine aggregates in equivalent proportions. Conversely, BF is expressed as the volume fraction of concrete. The specific proportions of the concrete mixture are displayed in Table 6.

### 2.3. Measurement Method

#### 2.3.1. Mechanical Properties

According to the Chinese code standard for testing methods of mechanical properties on ordinary concrete (GB/T 50081-2019) [30], the mechanical properties of concrete were tested. The size of the test block was 100 mm × 100 mm × 100 mm, and it was injected into the test molds after sufficient mixing by the mixer, with three test blocks for each group, with two groups for each proportion. The molds were taken off after 24 h, and the standard curing was carried out for up to 28 days. The two specimen groups were utilized for compressive strength and splitting tensile strength testing, respectively, and average strength values.

#### 2.3.2. Abrasion Resistance Test of Concrete

The study referred to the ring method in Chinese code: Test code for hydraulic concrete (SL/T 352-2020) [31]. The specimen was cured in the standard curing room (temperature 20 ± 2 °C, humidity more than 95%) for 26 days and then put into the water to soak for 2 days at constant weight to start the test. The operation of the machine was ceased after 30 min of abrasion and the specimen was simultaneously extracted, cleaned with water, the surface moisture was wiped off, and the specimen was weighed. After repeating the test 3 times for each specimen, abrasion resistance strength was calculated according to Equation (1) to characterize the abrasion resistance of concrete. The test result was determined by averaging the measured values of three specimens.

(1) $$f_{a}=\frac{TA}{\Delta M}$$

where *f_a_* represents the concrete’s abrasion resistance, which is the amount of time it takes to abrade a specific mass per unit area, measured in h(kg/m^2^)^−1^. *T* is the duration of the abrasion process, measured in h. *A* is the area of the specimen that is subjected to abrasion, specifically the inner ring area of the specimen, measured in m^2^. Δ*M* represents the mass loss after the abrasion process at time *T*, measured in kg.

#### 2.3.3. Microstructure Analyses

SEM analysis was conducted using a Hitachi SU-8010 electron microscope (HITACHI, Tokyo, Japan). Cubic specimens were subjected to compression testing with a hydraulic universal testing machine. Samples in flake form measuring 5 mm to 8 mm were selected, soaked in anhydrous ethanol, and subsequently dried. Prior to analysis, the sample surfaces were polished with sandpaper and treated with gold sputtering under vacuum conditions. XRD analysis was performed using a D/MAX 2000 X-ray diffractometer (HITACHI, Tokyo, Japan) to assess the physical phase composition of the specimens, operating at a tube pressure of 40 KV, tube current of 40 mA, and power of 1600 W, with a scanning angle of 5–80° and a scanning speed of 10°/min. The powder sample utilized in the XRD analysis was subjected to sieving through a 0.074 mm mesh and subsequently dried at 105 °C for 6 h prior to storage for future use.

## 3. Results and Analysis of Single Doping Test

### 3.1. Compressive Strength Analysis

**Figure 1:**
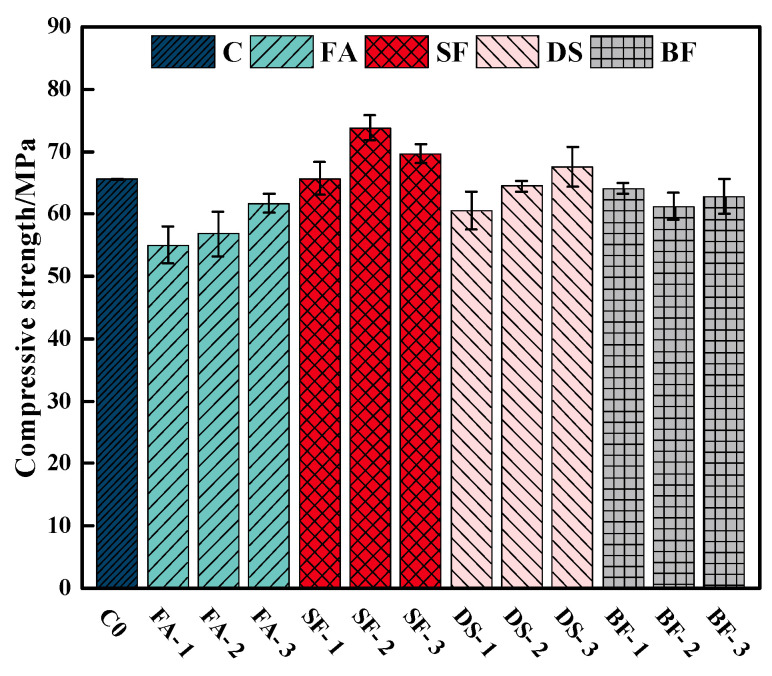
Impact of various admixtures on the 28 d compressive strength of concrete.

Figure 1 displays the test results of several admixtures on the 28 d compressive strength of concrete.

The substitution of cement with FA results in a decrease in the strength of concrete. Specifically, the 28 d compressive strength of concrete decreases by 16.16%, 13.41%, and 5.95% when the FA admixture is 10%, 20%, and 30%, respectively, compared to the unadulterated concrete. Yet, the compressive strength of FA-adulterated concrete gradually increases as the amount of FA admixture increases. The higher 28-day strength of pure cement concrete compared to FA-infused concrete is attributed to the lower activity and slower hydration rate of FA [32]. However, as the amount of FA admixture increases, it promotes the involvement of more reactive substances in the pozzolanic reaction and leads to the generation of more calcium silicate hydrate (C-S-H) gel [33]. This, in turn, enhances the strength of the concrete. Moghaddam et al. [34] proposed a comparable theory based on their research.

The inclusion of SF in concrete enhances its compressive strength. It is evident that when the amount of SF admixture increases, the strength of concrete exhibits a pattern of initially increasing and subsequently dropping. The highest compressive strength is achieved when the dosage of SF is 10%, resulting in a 12.50% increase in compressive strength compared to OC. The enhanced compressive strength of concrete due to the addition of SF can be attributed to the micro-aggregate effect and the pozzolanic effect of SF. The small particles of SF enhance compactness by occupying the empty spaces between the particles of the matrix. However, silicon dioxide (SiO_2_) in SF can chemically react with calcium hydroxide (CH), which is a cement hydration product, resulting in the formation of C-S-H gel. This gel enhances the interface bond strength between the concrete components [35], ultimately leading to an increase in the compressive strength of the concrete. Nevertheless, when the dosage of SF increases, there is a corresponding drop in the amount of cement, resulting in a decrease in the strength of the concrete. Luo et al. [36] also discovered in their research that when the SF dosage surpasses 10%, it leads to instability in the concrete, which has a negative impact on its strength.

The influence of DS on the compressive strength of concrete is determined by DSRR, which means that the compressive strength of concrete rises as DSRR increases. This corresponds to Rennani’s [37] research findings. When DS is added at a ratio of 60%, the compressive strength of the concrete increases by 2.90%. However, at 20% and 40% DSRR, the compressive strength of DSC was lower than that of OC, with a decrease of 7.77% and 1.68%, respectively. When the DSRR is low, the strength of DSC is inferior to that of OC due to the diminished strength of DS itself [38]. However, as the DSRR increases, the incorporation of DS enhances the fluidity of the concrete paste and significantly decreases the porosity of the concrete [39], positively influencing the enhancement of its mechanical properties. Furthermore, due to the presence of calcium oxide (CaO) and reactive aluminum trioxide (Al_2_O_3_) in DS, these components react with the hydration products of the cement, resulting in a pozzolanic reaction [40,41]. This reaction ultimately contributes to an enhancement in the compressive strength of the concrete.

The incorporation of BF did not significantly impact the enhancement of concrete’s compressive strength, mirroring the findings of Algin Zeynep’s research [42]. The reduction in the compressive strength of concrete results from the agglomeration of fibers inside the matrix, which elevates the porosity of the concrete and thus diminishes strength [43,44].

### 3.2. Splitting Tensile Strength Analysis

**Figure 2:**
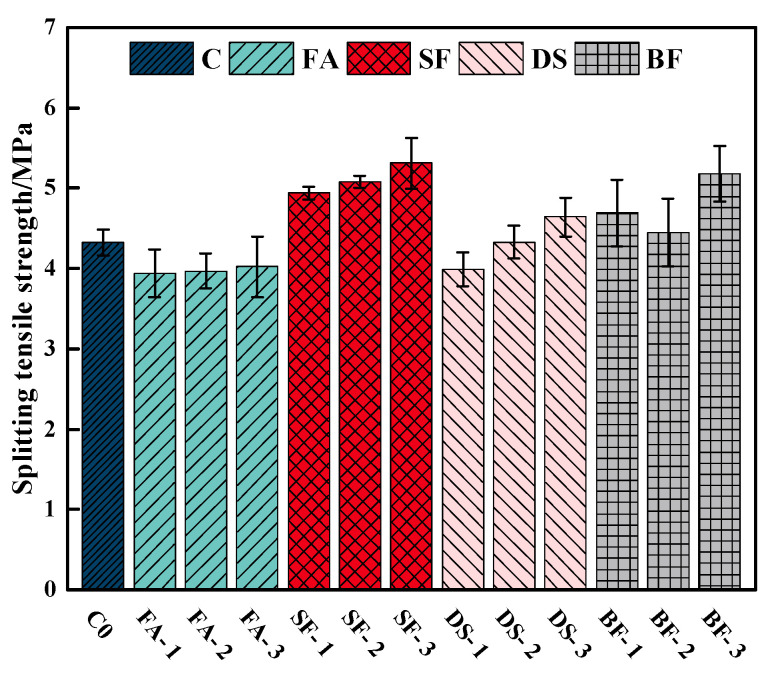
Impact of various admixtures on the 28 d splitting tensile strength of concrete.

Figure 2 displays the test results of several admixtures on the 28 d splitting tensile strength of concrete. The incorporation of FA results in a reduction in the splitting tensile strength of concrete. As the amount of FA admixture increases, there is only a slight improvement in the splitting tensile strength. This can be attributed to the lower reactant activity of FA, which hinders the formation of hydration products [45] and consequently reduces the splitting tensile strength of concrete.

The addition of SF admixture led to an increase in the splitting tensile strength of the concrete. Compared to OC, the splitting tensile strength increased by 14.35%, 17.59%, and 22.92%, respectively. This improvement can be attributed to the incorporation of SF, which enhances the compactness of the concrete and improves the interfacial effect between the cement paste and aggregates [46]. As a result, the splitting tensile strength of the concrete admixed with SF is superior to that of OC.

The variation pattern of the splitting tensile strength of DSC, as it relates to the increase in DSRR, aligns with the variation pattern observed in the compressive strength. The splitting tensile strength of the concrete reaches its peak when the DS admixture is at a content of 60%, resulting in a 7.41% increase compared to OC. In contrast to the compressive strength findings, the splitting tensile strength of DSC exhibited a modest increase at a DSRR of 40%.

Adding BF to concrete can improve its splitting tensile strength. The greatest splitting tensile strength of concrete is achieved when the fiber admixture is 0.15%. The main causes contributing to the enhancement of splitting tensile strength in concrete when fibers are added are the interfacial friction effect and spatial confinement effect [47].

### 3.3. Abrasion Resistance Test Analysis

**Figure 3:**
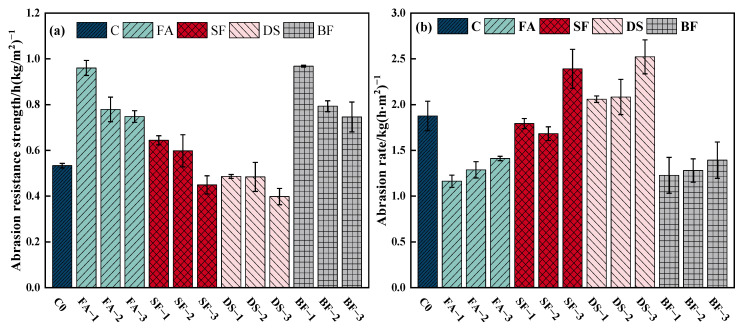
(**a**) Abrasion resistance strength, (**b**) abrasion rate.

The test results for the abrasion resistance strength and abrasion rate of concrete at 28 d, using various admixtures, are displayed in Figure 3a,b. The incorporation of SF in concrete can improve its abrasion resistance; however, the abrasion resistance strength of concrete consistently declines as the SF admixture increases. At SF admixtures of 5% and 10%, the concrete’s abrasion resistance strength increased by 20.82% and 12.27%, respectively, compared to OC. In addition, the abrasion rate was lower than that of OC. This improvement can be attributed to the pozzolanic effect of SF, which enhances the compactness of the concrete surface paste [48]. As a result, the quality loss due to abrasion is reduced. Nevertheless, if the proportion of SF exceeds 10%, increasing the amount of SF in the concrete would result in a more pronounced drying shrinkage phenomenon, making it more susceptible to cracking [49]. Furthermore, this will have a negative impact on the concrete’s abrasion resistance.

In comparison to OC, the abrasion resistance strength of concrete incorporated with DS (DSC) diminishes as the DSRR increases, decreasing by 8.88%, 9.15%, and 25.33% at DSRR levels of 20%, 40%, and 60%, respectively. Concurrently, the abrasion rate escalates with rising DSRR, increasing by 9.76%, 11.02%, and 34.45%, respectively. These results demonstrate that the addition of DS alone does not improve the abrasion resistance of concrete but instead leads to a decrease in it.

To summarize, the addition of FA and BF to concrete can enhance its abrasion resistance. Both admixtures exhibit the highest abrasion resistance strength and the lowest abrasion rate when used in small amounts. However, it is important to note the mechanisms by which these admixtures differ from each other. The addition of FA diminished the cement hydration heat and mitigated cracks caused by concrete shrinkage [50], hence enhancing its abrasion resistance. BF evenly distributes randomly within the concrete, forming a distinct three-dimensional network structure. This structure effectively prevents the creation and diffusion of cracks during the early stages of cement shrinkage and hardening [51]. It also reduces the detachment of fragments from the concrete matrix, thereby minimizing damage caused by abrasion.

**Figure 4:**
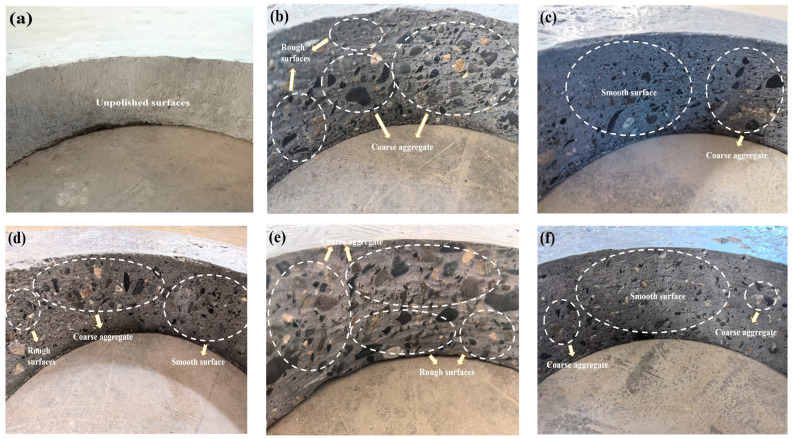
Concrete appearance prior to and subsequent to the completion of abrasion: (**a**) Prior to abrasion. (**b**) Following C0 abrasion. (**c**) Following FA-1 abrasion. (**d**) Following SF-2 abrasion. (**e**) Following DS-2 abrasion. (**f**) Following BF-1 abrasion.

Figure 4 displays the surface of the inner ring of concrete both before and after the abrasion resistance test. Figure 4a,b illustrates the surface of the inner ring prior to and subsequent to the abrasion of OC, respectively. The concrete surface was smooth and devoid of abrasions prior to abrasion. Figure 4c–f illustrates the inner wall morphology of the concrete following the execution of abrasion resistance tests on the concrete containing FA, SF, DS, and BF. The results indicate that the FA-1 group (with a 10% FA dosage), and the BF-1 group (with a 0.05% BF dosage) show minimal abrasion on the surface layer of the concrete. The inner ring surface remains smooth, with only a small portion of the coarse aggregate exposed.

Similarly, the C0 group (OC) and the SF-2 group (with a 10% SF dosage) exhibit similar levels of abrasion, with some of the coarse aggregate exposed. Both surfaces have a portion of the coarse aggregate visible, and a small section of the abrasion surface is not even. Nevertheless, the SF-2 (10% SF admixture) group had a minor area of smooth abrasion surface, whereas the C0 (OC) group displayed no smooth abrasion surface, indicating that the abrasion degree of the SF-2 group was less severe.

On the other hand, the inner ring surface of the DS-2 (DSRR of 40%) group experiences the most serious abrasion, with numerous coarse aggregates exposed and an uneven and rough abrasion surface. The surface exhibited irregularities and a coarse texture. Through the evaluation of the abrasion level on the inner ring surface of the test specimens, it was shown that the addition of FA and BF significantly enhanced the abrasion resistance of concrete. Conversely, the inclusion of DS had a detrimental effect on the abrasion resistance of concrete.

To summarize, when FA was mixed in at a concentration of 10% and BF at 0.05%, the concrete exhibited the highest abrasion resistance strength and the lowest abrasion rate. When SF was mixed at 10% and BF at 0.05%, the concrete showed the highest compressive strength. The addition of DS slightly improved the mechanical properties of the concrete but had a negative impact on its abrasion resistance. Hence, after careful consideration, the optimal combination for a single admixture consists of 10% FA content, 10% SF content, 40% DSRR, and 0.05% BF content, respectively.

### 3.4. Microanalysis

#### 3.4.1. SEM Analysis

**Figure 5:**
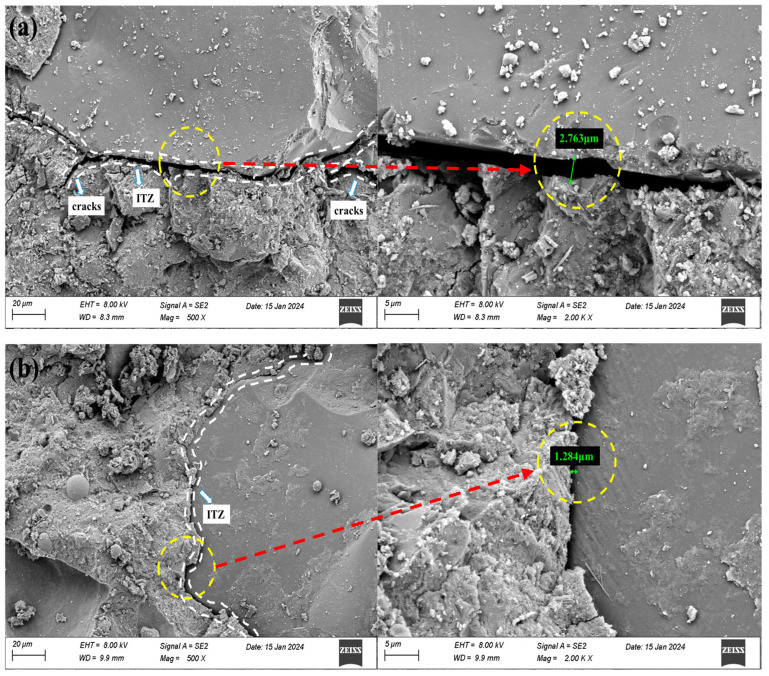

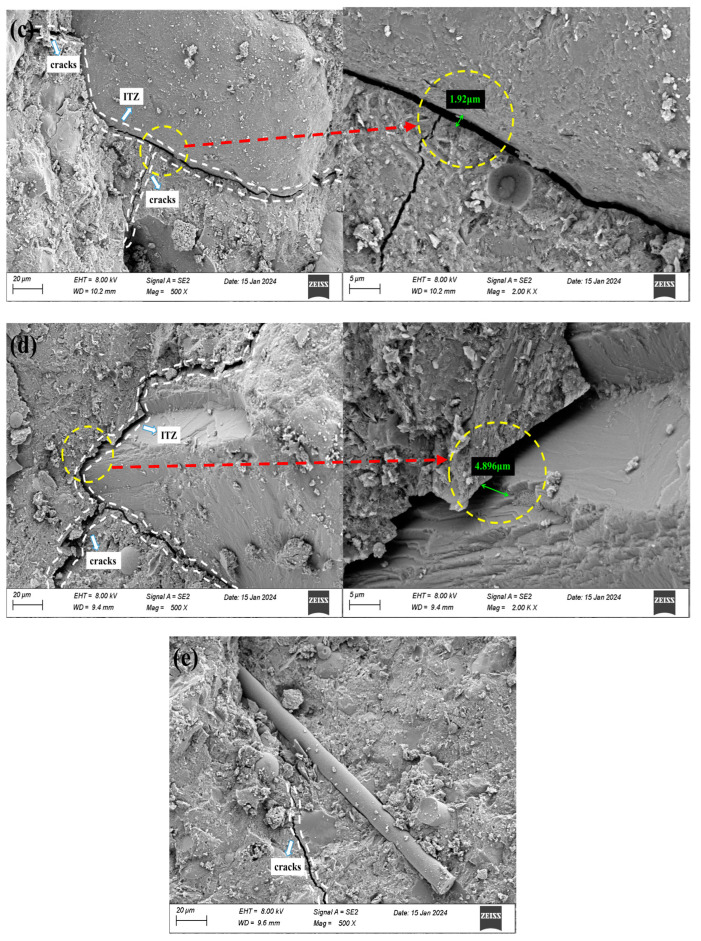
Concrete SEM microscopic morphology. (**a**) C0, (**b**) FA-1, (**c**) SF-2, (**d**) DS-2, and (**e**) BF-1.

The microscopic morphology of the concrete with the single optimal admixture was observed using SEM, and the representative results are presented in Figure 5. Figure 5a–c reveals that the OC specimens exhibit a broader interfacial transition zone (ITZ) measuring 2.763 μm alongside a limited number of microcracks and a looser structure. The inclusion of FA and SF markedly enhanced the interface bonding between aggregate and paste, resulting in ITZ widths lower than the 2.763 μm observed in OC, with fracture widths likewise finer than those. Figure 5b,c illustrates that the ITZ widths of concrete incorporating FA and SF are 1.284 μm and 1.92 μm, respectively. The ITZ of concrete with FA is narrower than that of concrete with SF. This microscopic interfacial structure partially reflects the outcomes of macroscopic testing, as a smaller ITZ width contributes to the paste’s resistance to scouring by water flow, thereby enhancing the concrete’s abrasion resistance strength. FA improves the abrasion resistance of concrete to a greater extent than SF. Comparing Figure 5a,d, it is observed that the ITZ width of DSC measures 4.896 μm, exceeding that of OC, and a looser structure and poor integrity in DSC. The soft and smooth surface of the DS may negatively influence the ITZ properties, leading to diminished abrasion resistance. Figure 5e illustrates that the introduction of BF into the concrete results in a random distribution of the fibers, lacking a defined arrangement, while the fiber surfaces are coated with numerous hydration products that enhance the adhesion of the fibers to the concrete matrix. The crack resistance role of the fibers restraining crack formation in the concrete leads to a denser structure of the fiber-reinforced concrete and enhanced abrasion resistance.

#### 3.4.2. XRD Analysis

**Figure 6:**
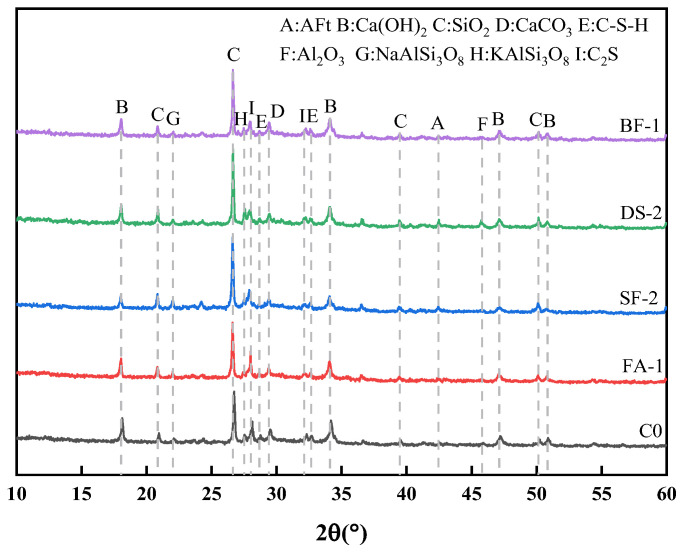
XRD patterns of several admixtures.

Figure 6 illustrates the XRD patterns of the hydration products produced from concrete using various admixtures. The incorporation of admixtures did not alter the primary crystalline phases of the hydration products, which predominantly included ettringite (AFt), Ca(OH)_2_, SiO_2_, calcium carbonate (CaCO_3_), C-S-H, and a minor presence of dicalcium silicate (C_2_S). Notably, C_2_S is the principal mineral in the cement [52], indicating that unhydrated cement clinker minerals remained in the concrete after 28 days of curing.

A notable Al_2_O_3_ diffraction peak is observed at 2θ = 45.813°, where Al_2_O_3_ can react with Ca(OH)_2_, a hydration product of cement, to produce hydrated calcium aluminates, thereby enhancing the compressive strength of concrete [53], consistent with the compressive strength results of concrete containing a macroscopic admixture of DS. The diffraction peak of orthoclase (KAlSi_3_O_8_) (2θ = 27.506°) exceeded that of OC, and the inclusion of aluminosilicates can engage in the hydration reaction of cement, producing additional sodium alumino-silicate hydrate (NASH) and calcium aluminosilicate hydrate (CASH) gels [54,55], thereby enhancing the strength of concrete. Consequently, there is a propensity to enhance the compressive strength of DSC.

## 4. Orthogonal Experiment

### 4.1. Design of Experiments

Orthogonal experiments were conducted to investigate the ideal mix proportion, taking into account the interaction among various admixtures. Based on a single doping optimum quantity, the FA dosage is set at ±5%, the SF dosage at ±2.5%, the DSRR at ±10%, and the BF dosage at ±0.025%. An orthogonal experiment comprising four factors and three levels, designated as L_9_(3^4^), was formulated. The experiment factors and levels are shown in Table 7, while the orthogonal experiment scheme is detailed in Table 8.

### 4.2. Results and Analyses of the Tests

#### 4.2.1. Experiment Outcomes

The test outcomes of DSC 28 days are presented in Table 9.

The concrete samples (1^#^–9^#^) containing FA, SF, DS, and BF exhibit superior abrasion resistance strength compared to the C0 group (OC). The orthogonal test for group 1^#^ exhibited the lowest abrasion resistance strength, but their abrasion resistance strength was still increased by 15.95% compared to that of the C0 group. This signifies that the synergistic effect of the four elements can significantly enhance the abrasion resistance of concrete. The superposition effect can explain this [24]; the pozzolanic effect and the micro-aggregate effect of FA, SF, and DS can enhance the compactness of concrete, while DS can create a continuous grading with river sand due to its small, rounded particle size. BF establishes a distinctive three-dimensional network structure in concrete, a configuration that obstructs the formation and propagation of fractures.

#### 4.2.2. Range Analysis

The primary and secondary sequences of components were ascertained using range analysis [56]. The range analysis data of DSC, considering FA content, SF content, DSRR, and BF content, is presented in Table 9, while the range analysis findings are displayed in Table 10.

In Table 10, *K_jm_* represents the aggregate of test indicators *y_jm_* associated with the *m* level in column factor *j*, with its computation delineated in Equation (2); *k_jm_* denotes the mean value of *K_jm_*, as articulated in Equation (3); and *R_j_* signifies the range of the *j*th column factor, defined as the disparity between the highest and lowest values of the indicator values across the levels of factors in the *j*th column, with its calculation detailed in Equations (4) and (5).

Utilizing the magnitude of *k_jm_*, one can ascertain the best level of factor *j* and the optimal combinations of each factor. The magnitude of *R_j_* can be utilized to ascertain the extent of variations in the test indicators when the quantity of the factors in the j column alters. The larger *R_j_* is, the more significant the factor’s impact on the test indicators, hence increasing the importance of its factors. Consequently, based on the magnitude of the range *R_j_*, the factors can be classified as principal or secondary.

(2) $$K_{jm}=\sum_{n=1}^{m}y_{jm}$$

(3) $$k_{jm}=\left(\sum_{n=1}^{m}K_{jm}\right)/m$$

(4) $$\mathrm{When}\;a\;\mathrm{greater}\;\mathrm{value}\;\mathrm{is}\;\mathrm{preferable},\;R_{j}=\max(k_{j1},k_{j2},\ldots ,k_{jm})-\min(k_{j1},k_{j2},\ldots ,k_{jm})$$

(5) $$\mathrm{When}\;\mathrm{lesser}\;\mathrm{values}\;\mathrm{are}\;\mathrm{preferable},\;R_{j}=\min(k_{j1},k_{j2},\ldots ,k_{jm})-\max(k_{j1},k_{j2},\ldots ,k_{jm})$$

The data in Table 10 indicates that the influence of each factor on the compressive strength of concrete is ranked as follows: FA > BF > SF > DS, corresponding to A > D > B > C. The optimal mix ratio based on the maximum compressive strength of concrete is A1B2C2D2. The significant and minimal impact of each factor on the splitting tensile strength of concrete is as follows: SF > BF > FA > DS, or B > D > A > C, which results in the best mix ratio for maximum splitting tensile strength of concrete, which is A2B3C2D3. The influence of each factor on the abrasion resistance strength of concrete is ranked as follows: FA > DS > SF > BF, corresponding to A > C > B > D. The optimal mix ratio for achieving maximum abrasion resistance strength in concrete is A2B2C2D2. The relative influence of each element on the concrete abrasion rate is as follows: BF > SF > DS > FA, corresponding to D > B > C > A. The ideal blend ratio for minimizing concrete abrasion rate is A2B2C2D2.

In conclusion, the study’s principal findings reveal that the ideal mix ratio for DSC, with respect to the abrasion resistance of concrete, is A2B2C2D2, including 10% FA content, 10% SF content, 40% DSRR, and 0.05% BF content.

#### 4.2.3. Variance Analysis

Range analysis is straightforward, although fluctuations in levels and unpredictability may result in errors in the test outcomes, whereas variance analysis enhances the precision of the results and mitigates errors [57]. The variance analysis findings are presented in Table 11.

Table 11 indicates that FA, SF, and BF significantly influence the compressive strength of concrete, whereas DS has an insignificant effect. FA, SF, DS, and BF all significantly affect the splitting tensile strength of concrete. The influence of FA, SF, and DS on the abrasion resistance strength of concrete was substantial, whereas the effect of BF was negligible; however, BF significantly affected the abrasion rate of concrete, followed by SF, while FA and DS had negligible effects, aligning with the findings of the range analysis.

#### 4.2.4. Analysis of Factor Indicators

**Figure 7:**
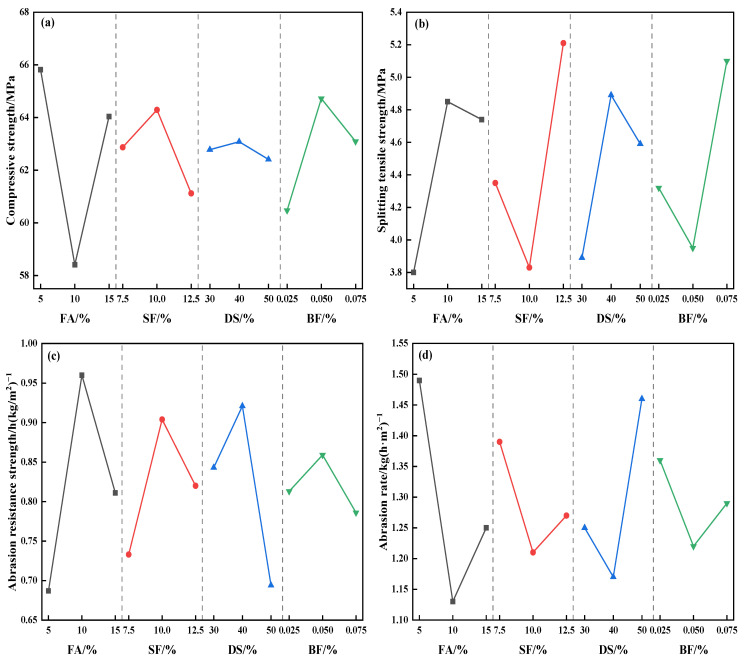
Variation trends of each factor at different levels. (**a**) Compressive strength, (**b**) splitting tensile strength, (**c**) abrasion resistance strength, (**d**) abrasion rate.

Figure 7 illustrates the trend of each factor across several levels. Figure 7a demonstrates that the compressive strength of concrete initially declines and subsequently increases with the rising amount of FA. The compressive strength of concrete attained its maximum at an FA content of 5%. The compressive strength of concrete exhibited an initial increase, subsequently followed by a decline, with varying contents of SF, DS, and BF. The maximum compressive strength occurred at 10% SF content, 40% DSRR, and 0.05% BF content, indicating the inflection point of strength fluctuation.

Figure 7b illustrates that the concrete splitting tensile strength initially increases and subsequently decreases with rising contents of FA and DS, reaching its peak at 10% FA content and 40% DSRR. The concrete splitting tensile strength had a decreasing trend, subsequently accompanied by a rising pattern with the enhancement of SF and BF contents, reaching its maximum at 12.5% SF content and 0.075% BF content.

Figure 7c demonstrates that the abrasion resistance strength of concrete initially increases and then subsequently decreases when the proportions of FA, SF, DS, and BF increase. The optimal abrasion resistance strength is attained with an FA content of 10%, SF content of 10%, DSRR of 40%, and BF content of 0.05%.

Figure 7d reveals that the concrete abrasion rate initially decreases and increases afterward when the contents of FA, SF, DS, and BF increase. The minimum abrasion rate occurs at an FA content of 10%, SF content of 10%, DSRR of 40%, and BF content of 0.05%. This corresponds to the trend of concrete’s abrasion resistance strength.

### 4.3. Determination of Concrete Mixture Proportions

The conclusive mix proportion derived from the orthogonal experiment is A2B2C2D2, comprising 10% FA content, 10% SF content, 40% DSRR, and 0.05% BF content. This mix proportion was excluded from the nine sets of mix proportions in the orthogonal experiment. Consequently, it is imperative to retest this mix proportion to confirm its exactness. The test outcomes for the DSC 28 days are offered in Table 12.

Table 12 indicates that the compressive strength of the DSC-1 group is equivalent to that of the C0 (OC) group, satisfying the strength criteria for C50-grade concrete. The splitting tensile strength and abrasion resistance strength of the DSC-1 group surpass those of the C0 group, exhibiting increases of 39.12 percent and 112.95 percent, respectively; additionally, the abrasion rate of the DSC-1 group is the lowest, being 48.83 percent lower than that of the C0 group.

**Figure 8:**
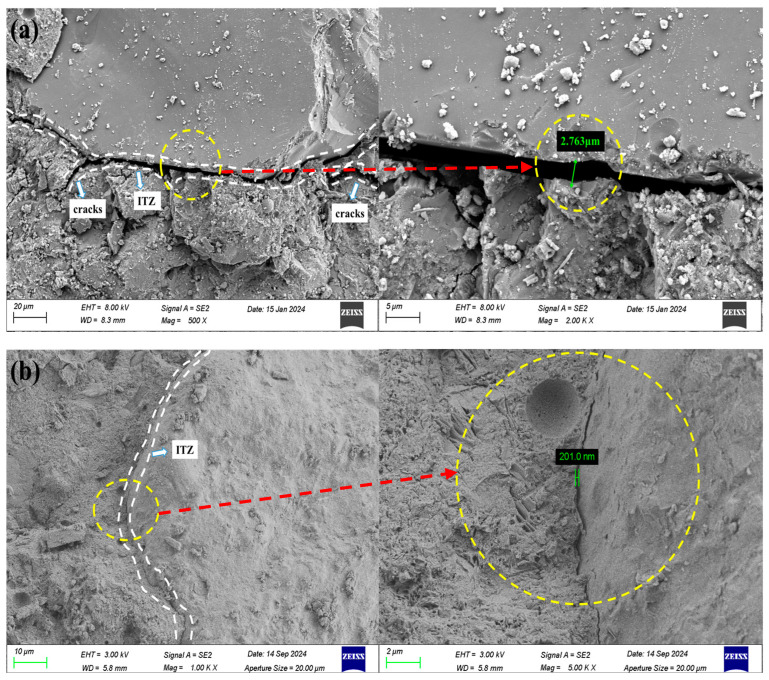
Micro-morphology of concrete SEM. (**a**) Group C0. (**b**) Group DSC-1.

Figure 8a,b illustrate the microscopic morphology of the concrete from group C0 and group DSC-1, respectively. The concrete of the DSC-1 group has a dense structure with a narrow ITZ of 201.0 nm, in contrast to the C0 group. The microscopic features of the DSC-1 group indicate its superior abrasion resistance, with these microscopic phenomena aligning with the macroscopic results of abrasion resistance strength.

## 5. Discussion

Currently, the scarcity of river sand resources is making the utilization of DS in construction materials increasingly promising. The investigation of the performance variation in DSC in actual service conditions holds significant scientific worth. Nevertheless, the low fineness modulus of DS will influence the performance of concrete, including workability, strength, and durability. Consequently, enhancing the performance of DSC necessitates the use of additional components.

This study examined the influence of DS, FA, SF, and BF on the mechanical properties and the abrasion resistance of concrete, ultimately identifying the best DSC mix ratio with superior abrasion resistance using orthogonal experiments. The test findings indicate that the synergistic interaction of the four materials significantly enhances the mechanical properties and the abrasion resistance of concrete. Microscopic analysis investigation revealed that the breadth of the interfacial transition zone significantly influences the abrasion resistance of concrete.

Currently, numerous researchers have conducted pertinent studies on DSC. Li et al. [58] determined that the workability and mechanical properties of DSC satisfy the practical requirements of engineering. Other scholars [40,59,60] determined that concrete performance is optimal at a DSRR of 40%, aligning with the findings of this paper. Nevertheless, the abrasion resistance of concrete incorporating DS is unreported.

The findings of this study apply to the practical application of concrete channel engineering projects in desert areas, which facilitates the use of local materials and minimizes production expenses. In implementing the research findings from this test in practical engineering contexts, several issues need to be considered, including the on-site curing conditions of concrete and the durability of DSC in specific environments, such as frost resistance, impermeability, and corrosion resistance.

## 6. Conclusions

Through the individual incorporation of DS, FA, SF, and BF, an examination was conducted on their influence on the mechanical properties and abrasion resistance of concrete. Orthogonal experiments were devised to assess the effects of these four factors, leading to the following conclusions.

(1) The incorporation of FA and BF into concrete significantly enhances its abrasion resistance, with fewer admixture levels of FA and BF yielding superior performance in both aspects. When the SF dosage is below 10%, it enhances the abrasion resistance of concrete; however, the DS adversely influences the abrasion resistance of concrete.
(2) Orthogonal experiment findings indicated that the four admixtures collectively enhance the abrasion resistance of concrete. Variance analysis revealed that FA, SF, and DS significantly affect the abrasion resistance strength of concrete, but SF and BF significantly influence the abrasion rate of concrete.
(3) The analysis reveals that the ideal mix proportion for the DSC-1 group is A2B2C2D2, comprising 10% FA content, 10% SF content, 40% DSRR, and 0.05% BF content. Its compressive strength is comparable to that of OC; however, the splitting tensile strength and abrasion resistance strength of the DSC-1 group increased by 39.12% and 112.95%, respectively, compared to OC, while the abrasion rate decreased by 48.83%. This indicates that DS can be effectively utilized, hence diminishing reliance on river sand.
(4) Through the microscopic analysis, it is found that the width of the ITZ is an important factor in determining the abrasion resistance of concrete, and a narrower ITZ enhances the concrete’s abrasion resistance. The findings of this study may serve as a reference for the utilization of DSC in assembled concrete channels.

The findings indicate that the synergistic interaction between DS and mineral admixtures (FA, SF, and BF) markedly enhances the mechanical properties and the abrasion resistance of concrete. This not only offers a viable scheme for the efficient use of DS resources but also facilitates the usage of locally available materials, particularly in desert fringe regions, thereby substantially reducing concrete production costs. This study offers significant data to support the utilization of DS in engineering and construction within desert regions and adjacent areas, serving as a reference for the application of DSC in assembled channels. However, the constraints of orthogonal experiments preclude a comprehensive analysis of the interaction and synergy mechanisms among materials. Nevertheless, the study’s findings can still offer robust data to support the application of concrete materials in the field of construction engineering and sustainable development and can also serve as a reference for engineers in selecting more economically viable raw materials for practical projects, particularly in construction projects in desert and arid regions. The use of DS and mineral admixtures as concrete raw materials can diminish the carbon footprint and resource consumption of the building sector, which is advantageous for environmental conservation.

## Figures and Tables

**Table 1:** Chemical composition and specific surface area of cement.

Chemical Composition/%									Specific Surface Area/(m^2^/kg)
SiO_2_	CaO	Al_2_O_3_	MgO	SO_3_	Na_2_O	K_2_O	Fe_2_O_3_	Others	
19.94	62.40	5.26	3.78	3.00	0.13	1.00	2.98	1.51	348

**Table 2:** Chemical composition of Gurbantunggut Desert sand %.

SiO_2_	Al_2_O_3_	Fe_2_O_3_	Na_2_O	CaO	K_2_O	MnO	FeO	TiO_2_	P_2_O_5_	MnO
67.1%	17.9%	1.35%	4.94%	4.22%	3.48%	0.84%	0.06%	0.02%	0.05%	0.04%

**Table 3:** Fly ash parameters.

Hierarchy	Fineness (%)	Ignition Loss (%)	Water Demand Ratio (%)	Moisture Content (%)	Specific Surface Area/(m^2^/kg)
Ⅰ class	7.7	2.1	94	0.3	384

**Table 4:** Silica fume parameters.

SiO_2_(%)	Scorch Reduction (%)	Specific Surface Area (m^2^/g)	Chloride Ion (%)
96.2	3.92	19.1	0.07

**Table 5:** Fiber performance indicators.

Fiber Type	Length (mm)	Diameter (μm)	Density (g/cm^3^)	Breaking Elongation (%)	Drawing Strength (MPa)	Elasticity Modulus (GPa)
Basalt fiber	18	15	2.65	2.8	4000	105

**Table 6:** Mix proportion (kg/m^3^).

Test Number	Cement	Water	Fine Aggregate	Coarse Aggregate	Water Reducing Agent	FA	SF	DS	BF
C0	451.29	176	621.38	1201.34	4.52	0	0	0	0
FA-1	406.16	176	621.38	1201.34	4.52	45.13	0	0	0
FA-2	361.03	176	621.38	1201.34	4.52	90.26	0	0	0
FA-3	315.90	176	621.38	1201.34	4.52	135.39	0	0	0
SF-1	428.73	176	621.38	1201.34	4.52	0	22.56	0	0
SF-2	406.16	176	621.38	1201.34	4.52	0	45.13	0	0
SF-3	383.60	176	621.38	1201.34	4.52	0	67.69	0	0
DS-1	451.29	176	497.10	1201.34	4.52	0	0	124.28	0
DS-2	451.29	176	372.83	1201.34	4.52	0	0	248.55	0
DS-3	451.29	176	248.55	1201.34	4.52	0	0	372.83	0
BF-1	451.29	176	621.38	1201.34	4.52	0	0	0	1.33
BF-2	451.29	176	621.38	1201.34	4.52	0	0	0	2.65
BF-3	451.29	176	621.38	1201.34	4.52	0	0	0	3.98

**Table 7:** Table of experimental factor levels.

Level	Factor			
	FA Content A/wt%	SF Content B/wt%	DSRR C/wt%	BF Content D/vol%
1	5	7.5	30	0.025
2	10	10	40	0.05
3	15	12.5	50	0.075

Note: wt% denotes the mass percentage, while vol% signifies the volume fraction.

**Table 8:** Orthogonal experiment scheme (kg/m^3^).

Test Number	Cement	Water	Fine Aggregate	Coarse Aggregate	Water-Reducing Agent	FA	SF	DS	BF
1^#^	394.88	176	434.96	1201.34	10.38	22.56	33.85	186.41	0.66
2^#^	383.60	176	310.69	1201.34	10.38	22.56	45.13	310.69	1.33
3^#^	372.31	176	372.83	1201.34	10.38	22.56	56.41	248.55	1.99
4^#^	372.31	176	310.69	1201.34	10.38	45.13	33.85	310.69	1.99
5^#^	361.03	176	372.83	1201.34	10.38	45.13	45.13	248.55	0.66
6^#^	349.75	176	434.96	1201.34	10.38	45.13	56.41	186.41	1.33
7^#^	349.75	176	372.83	1201.34	10.38	67.69	33.85	248.55	1.33
8^#^	338.47	176	434.96	1201.34	10.38	67.69	45.13	186.41	1.99
9^#^	327.19	176	310.69	1201.34	10.38	67.69	56.41	310.69	0.66

**Table 9:** Results of the concrete orthogonal experiment.

Test Number	Compressive Strength/MPa	Splitting Tensile Strength/MPa	Abrasion Resistance Strength/h (kg/m^2^)^−1^	Abrasion Rate/kg (h·m^2^)^−1^
1^#^	63.67	2.98	0.618	1.62
2^#^	68.97	2.79	0.686	1.50
3^#^	64.83	5.62	0.756	1.34
4^#^	58.50	5.51	0.715	1.40
5^#^	57.97	4.51	1.140	1.00
6^#^	58.75	4.52	1.024	0.99
7^#^	66.43	4.55	0.866	1.16
8^#^	65.93	4.18	0.887	1.13
9^#^	59.77	5.48	0.680	1.47

**Table 10:** Results of range analysis.

Test	Index	Level	Factor			
			A	B	C	D
Compressive Strength/MPa	*K_jm_*	1	197.47	188.6	188.35	181.41
		2	175.22	192.87	189.23	194.15
		3	192.13	183.35	187.24	189.26
	*k_jm_*	1	65.82	62.87	62.78	60.47
		2	58.41	64.29	63.08	64.72
		3	64.04	61.12	62.41	63.09
	Optimal Level		1	2	2	2
	*R_j_*		7.42	3.17	0.66	4.25
Splitting Tensile Strength/MPa	*K_jm_*	1	11.39	13.04	11.68	12.97
		2	14.54	11.48	14.68	11.86
		3	14.21	15.62	13.78	15.31
	*k_jm_*	1	3.80	4.35	3.89	4.32
		2	4.85	3.83	4.89	3.95
		3	4.74	5.21	4.59	5.10
	Optimal Level		2	3	2	3
	*R_j_*		1.05	1.38	1	1.15
Abrasion Resistance Strength /h(kg/m^2^)^−1^	*K_jm_*	1	2.060	2.199	2.529	2.438
		2	2.879	2.713	2.762	2.576
		3	2.433	2.460	2.081	2.358
	*k_jm_*	1	0.687	0.733	0.843	0.813
		2	0.960	0.904	0.921	0.859
		3	0.811	0.820	0.694	0.786
	Optimal Level		2	2	2	2
	*R_j_*		0.273	0.171	0.227	0.073
Abrasion Rate /kg(h·m^2^)^−1^	*K_jm_*	1	4.46	4.18	3.74	4.09
		2	3.39	3.63	3.50	3.65
		3	3.76	3.80	4.37	3.87
	*k_jm_*	1	1.49	1.39	1.25	1.36
		2	1.13	1.21	1.17	1.22
		3	1.25	1.27	1.46	1.29
	Optimal Level		2	2	2	2
	*R_j_*		−0.36	−0.18	−0.29	−0.14

**Table 11:** Results of variance analysis.

Test	Factor	Quadratic Sum	Degree of Freedom	Mean Square	*F*	*p*
Compressive Strength /MPa	A	267.294	2	133.647	122.480	0.000 **
	B	52.817	2	26.409	24.202	0.000 **
	C	4.984	2	2.492	2.284	0.131
	D	70.745	2	35.373	32.417	0.000 **
Splitting Tensile Strength /MPa	A	5.497	2	2.748	23.257	0.000 **
	B	8.210	2	4.105	34.739	0.000 **
	C	5.376	2	2.688	22.748	0.000 **
	D	6.182	2	3.091	26.158	0.000 **
Abrasion Resistance Strength /h(kg/m^2^)^−1^	A	3.290	2	1.645	0.757	0.0004 **
	B	1.365	2	0.682	0.314	0.0068 **
	C	2.464	2	1.232	0.567	0.0012 **
	D	0.262	2	0.131	0.060	0.0558
Abrasion Rate /kg(h·m^2^)^−1^	A	0.01	2	0.00	19.80	1.67
	B	0.04	2	0.02	77.27	0.04 *
	C	0.02	2	0.01	30.18	0.74
	D	0.06	2	0.03	121.28	0.00 **

Note: * *p* < 0.05, essential but ** *p* < 0.01, extremely important.

**Table 12:** Results of the final mix proportion test.

Test Number	Compressive Strength/MPa	Splitting Tensile Strength/MPa	Abrasion Resistance Strength/h(kg/m^2^)^−1^	Abrasion Rate/kg(h·m^2^)^−1^
C0	65.6	4.32	0.533	1.876
DSC-1	65.93	6.01	1.135	0.96

## Data Availability

The original contributions presented in this study are included in the article. Further inquiries can be directed to the corresponding authors.
